# Phospholipase D affects translocation of NPR1 to the nucleus in *Arabidopsis thaliana*

**DOI:** 10.3389/fpls.2015.00059

**Published:** 2015-02-18

**Authors:** Martin Janda, Vladimír Šašek, Hana Chmelařová, Jan Andrejch, Miroslava Nováková, Jana Hajšlová, Lenka Burketová, Olga Valentová

**Affiliations:** ^1^Department of Biochemistry and Microbiology, University of Chemistry and Technology PraguePrague, Czech Republic; ^2^Laboratory of Pathological Plant Physiology, Institute of Experimental Botany AS CRPrague, Czech Republic; ^3^Department of Food Analysis and Nutrition, University of Chemistry and Technology PraguePrague, Czech Republic

**Keywords:** *n*-butanol, NPR1, salicylic acid, phospholipase D, signaling, *PR-1*, metabolome

## Abstract

Phytohormone salicylic acid (SA) is a crucial component of plant-induced defense against biotrophic pathogens. Although the key players of the SA pathway are known, there are still gaps in the understanding of the molecular mechanism and the regulation of particular steps. In our previous research, we showed in *Arabidopsis* suspension cells that *n*-butanol, which specifically modulates phospholipase D activity, significantly suppresses the transcription of the pathogenesis related (*PR-1*) gene, which is generally accepted as the SA pathway marker. In the presented study, we have investigated the site of *n*-butanol action in the SA pathway. We were able to show in *Arabidopsis* plants treated with SA that *n*-butanol inhibits the transcription of defense genes (*PR-1, WRKY38*). Fluorescence microscopy of *Arabidopsis thaliana* mutants expressing *35S::NPR1-GFP* (nonexpressor pathogenesis related 1) revealed significantly decreased nuclear localization of NPR1 in the presence of *n*-butanol. On the other hand, *n*-butanol did not decrease the nuclear localization of NPR1 in *35S::npr1C82A-GFP* and *35S::npr1C216A-GFP* mutants constitutively expressing NPR1 monomers. Mass spectrometric analysis of plant extracts showed that *n*-butanol significantly changes the metabolic fingerprinting while *t*-butanol had no effect. We found groups of the plant metabolites, influenced differently by SA and *n*-butanol treatment. Thus, we proposed several metabolites as markers for *n*-butanol action.

## Introduction

The resistance of plants to pathogens relies on a sophisticated immune system comprising an orchestra of defense mechanisms. The efficiency is highly dependent on the speed of the process starting with pathogen recognition and resulting in the expression of appropriate defense proteins.

Salicylic acid (SA) is a crucial phytohormone involved in the defense response mostly to biotrophs (Glazebrook, 2005; Tsuda et al., 2008; Tsuda and Katagiri, 2010), but several reports on the defense against necrotrophs also exist (Novakova et al., 2014). The key enzyme in SA biosynthesis is isochorismate synthase (ICS; EC 5.4.4.2) that catalyses the conversion of chorismate into isochorismate. ICS is encoded by two genes in *Arabidopis thaliana*. This pathway has been shown to be the dominant SA biosynthetic pathway in response to attack by pathogenic bacteria, contributing to approximately 90% of total SA, with most ICS activity attributed to ICS1 and ICS2, which ICS2 plays only a marginal role (Wildermuth et al., 2001). SA is catabolized in infected and senescing plants by the recently found enzyme salicylic acid-3-hydroxylase (S3H), which catalyzes conversion of SA to 2,3-dihydroxybenzoic acid (2,3-DHBA; gentisic acid) and thus regulates the level of SA in plants (Zhang et al., 2013). The SA mode of action has been intensively studied for more than 20 years (Vlot et al., 2009). The crucial component of the SA pathway is a nonexpressor of pathogenesis related 1 (NPR1) protein (Cao et al., 1994). It was shown that NPR1 influences transcription of ~90% of the SA dependent defense genes (Wang et al., 2005; Blanco et al., 2009). In cytosol, NPR1 occurs as an oligomer. Increased amounts of SA cause the monomerization of the NPR1 oligomer due to the change of the redox state in the plant cell (Mou et al., 2003). Thereafter, the NPR1 monomer is translocated to the nucleus where the NPR1 monomers bind to the TGA transcription factors followed by their direct binding to the as-1 (activation sequence 1) cis-regulatory element that is present in the promoters of *PR* (pathogenesis related) genes, thus activating their expression (Jakoby et al., 2002). The *PR-1* gene is generally accepted as the marker for SA signaling. The monomeric NPR1, in the nucleus, is continuously degraded by proteasome, a process which plays a dual function in the induction of transcription of the SA related genes (e.g., *PR-1*) (Wang et al., 2005; Spoel et al., 2009). Proteasome degradation lowers the amount of NPR1 in the nucleus, but considering that newly formed NPR1 is needed for the induction of *PR-1* transcription, the proteasome plays a key role in the regulation of NPR1 turnover (Spoel et al., 2009). Recently, a crucial step forward was made in the understanding of SA awareness; the long sought after SA receptor was probably found. Xinnian Dong's group showed that NPR3 and NPR4 (two orthologs of NPR1) have a binding affinity to SA. Interestingly, the binding affinity of NPR4 is much higher than that of NPR3, but this property is crucial for the correct regulation of NPR1 degradation and SA awareness (Fu et al., 2012).

Currently, it seems more obvious that the SA pathway is connected with the phospholipid signaling system (Janda et al., 2013), but the details are unknown. One of the key players of the phospholipid signaling in plants is phosphatidic acid (PA), produced by the action of phospholipase C and DAG kinase or directly by phospholipase D (PLD) (EC 3.1.4.4). PLD activity is specifically modulated by *n*-butanol due to the unique transphosphatidylation reaction catalyzed by this enzyme (Yang et al., 1967; Munnik et al., 1995). In the presence of low concentrations of primary alcohols, the phosphatidate moiety is preferentially transferred to the alcohol hydroxyl group rather than to the water molecule and the products of this reaction—phosphatidylalcohols are metabolically stable (Liscovitch et al., 2000). PLD occurs in *A. thaliana* in 12 isoforms with distinct biochemical and structural properties (Pleskot et al., 2012). Activation or increased expression of PLD isoforms after infection was shown in rice (Young et al., 1996; Lee et al., 1997; McGee et al., 2003) and *A. thaliana* (De Torres Zabela et al., 2002). The treatment with SA increased the PA level or PLD activity in *A. thaliana, Brassica napus* and soybean (Profotova et al., 2006; Kalachova et al., 2012; Rainteau et al., 2012). Zhao et al. (2013) investigated the role of AtPLDβ 1 in defense responses to bacterial pathogens. PLDβ1-deficient plants were less susceptible to *Pseudomonas syringae* and the transcription of SA responsive genes rose in infected plants compared to the wild-type infected plants (Zhao et al., 2013). Krinke et al. (2009) described that in *A. thaliana* suspension cells, *n*-butanol blocked the *PR-1* transcription in the presence of SA. However, the mechanism of PLD/PA involvement in SA signaling remains unclear.

This work provides evidence that *n*-butanol, the most effective primary alcohol modulating the activity of PLD, is involved in the regulation of *PR-1* transcription in the seedlings of *A. thaliana*. We show also that its action proceeds or participates in the process of NPR1 transfer to the nucleus. The non-targeted metabolomic fingerprinting provides evidence that *n*-butanol has a substantial impact on metabolome whereas *t*-butanol remains ineffective.

## Materials and methods

### Plant material

Seedlings of *A. thaliana* ecotype Col-0 (WT), and transgenic plants *35S::NPR1-GFP, 35S::npr1C82A-GFP, 35S::npr1C216A-GFP* (Kinkema et al., 2000; Mou et al., 2003) were grown in 24-well plates in 400 μL of MS liquid medium (Clay et al., 2009) for 10 days in a cycle of 10 h days (120 μE m^−2^ s^−1^, 22°C) and 14 h nights (22°C) at 70% relative humidity. MS liquid medium in the wells was changed on the 7th day.

### Chemical treatments

The plants were treated directly in the wells of plates by changing the growing medium for the chemical-containing medium. 10-day-old seedlings were treated for 6 h with 50 μM and 250 μM salicylic acid sodium salt (Sigma; NaSA), 0.1 and 1% *n*-butanol (Sigma) or *t*-butanol (Penta).

### Gene transcription analysis

The whole seedlings from three wells were immediately frozen in liquid nitrogen. The tissue was homogenized in tubes with 1 g of 1.3 mm silica beads using a FastPrep-24 instrument (MP Biomedicals, CA, USA). RNA isolation and reverse transcription were performed as previously described (Sasek et al., 2012). An equivalent of 6.25 ng of RNA was loaded into a 10 μl reaction with qPCR mastermix EvaLine—E1LC (GeneOn, Ludwigshafen am Rhein, Germany). The reactions were performed in polycarbonate capillaries (Genaxxon, Ulm, Germany) and a LightCycler 1.5 (Roche). The following PCR program was used for PCR assays: 95°C for 10 min; 45 cycles: 95°C for 10 s, 55°C for 10 s, and 72°C for 10 s; finished with a melting curve analysis. Threshold cycles and melting curves were calculated using LightCycler Software 4.1 (Roche). Alternatively, the LightCycler® 480 SYBR Green I master kit was used. The reactions were performed in the LightCycler® 480 Multiwell Plate 96 white. The following PCR program was used for PCR assays: 95°C for 10 min; 45 cycles: 95°C for 20 s, 55°C for 20 s, and 72°C for 10 s; finished with a melting curve analysis. The threshold cycles and melting curves were calculated using LightCycler Software 4.2 (Roche). The relative transcription was calculated with the efficiency correction and normalization (Czechowski et al., 2005). The primers were designed using PerlPrimer v1.1.17 (Marshall, 2004). The list of *A. thaliana* genes and corresponding accession numbers and primers follows: *SAND*, AT2G28390, FP: 5′CTG TCT TCT CAT CTC TTG TC 3′, RP: 5′ TCT TGC AAT ATG GTT CCT G 3′, *PR-1*, AT2G14610, FP: 5′ AGT TGT TTG GAG AAA GTC AG 3′, RP: 5′ GTT CAC ATA ATT CCC ACG A, *S3H*, AT4G10500, FP: 5′GGA TGA TAA ATG GGT CGC T 3′, RP: 5′TGT TTA CTA CGG CTC TAT GG 3′; *WRKY38*, AT5G22570, FP: 5′GCC CCT CCA AGA AAA GAA AG 3′, RP: 5′ CCT CCA AAG ATA CCC GTC GT 3′, *ICS1* AT1G74710 FP: 5′GCA AGA ATC ATG TTC CTA CC 3′, RP: 5′AAT TAT CCT GCT GTT ACG AG 3′.

### Confocal microscopy

The slide with seedlings was positioned onto an inverted platform (with a cover slip at the bottom) of the confocal laser scanning Zeiss LSM 5 DUO microscope. The GFP fluorescence was excited by the 488 nm line of a laser, the DAPI fluorescence was excited by the 405 nm line. The epidermal cells were viewed using an Zeiss Plan-Apochromat 20x/0,8 objective. The emitted light was captured using the HFT405/488 beam splitter and a 505–550 nm or 420–480 nm band-pass filter, respectively. Image analysis was performed using the software APS Asess 2.0.

### Metabolomic screening

The extraction procedure was modified according to Vaclavik et al. (2013). Whole seedlings from three independent wells were immediately frozen using liquid nitrogen. Six independent samples for one type of treatment were prepared for one biological replicate. 150–250 mg of plant tissue was homogenized in tubes with 1 g of 1.3 mm silica beads using a FastPrep-24 instrument (MP Biomedicals, CA, USA). After the addition of 700 μL of methanol (p.a.; PENTA), the plant tissue was homogenized again. The silica beads were washed once with 700 μL methanol and both extracts were combined. The samples were kept on ice during the extraction. Prior to instrumental analysis, the samples were stored in a dark and dry environment at −70°C. The UHPLC–Q-TOF-MS analyses were performed using an Acquity Ultra-Performance LC system coupled to a Synapt G2 high definition mass spectrometer (Waters, USA). The LC separation was performed by an Acquity UPLC® HSS T3 column (100 × 1.8 mm, 1.7 μm particle size; Waters, USA). The gradient elution was used with the mobile phases consisting of (A) 0.1% formic acid in Milli-Q water and (B) 0.1% formic acid in methanol.

The Synapt G2 HD instrument was operated in the negative electrospray ionization (ESI) mode. The parameter settings used during the measurements were as follows: capillary voltage (−700 V), cone voltage (−25 V), source temperature (120°C), and desolvation temperature (350°C). Nitrogen was used as both desolvation and cone gas at a flow rate of 800 and 10 L/h, respectively. Both full MS and MS/MS fragmentation mass spectra were acquired at a rate of two spectra per second in the range *m/z* 50–1000. In order to diminish any possible time dependent changes in the UHPLC-MS chromatographic fingerprints, the sequence of the samples was randomized and one sample was chosen as a quality control sample, which was injected after every set of 20 samples. The MassLynx 4.1 software (Waters, USA) was used for data acquisition and the MarkerLynx software (Waters, USA) was used for data mining and processing. The software SIMCA (v. 13.0, Umetrics, Sweden) was then used for data processing based on Principal Components Analysis (PCA).

### Data evaluation

Values are expressed as means ± standard error (SE). For statistical analysis, Student's *t*-test or One-Way ANOVA followed by Fisher's Least-Significant-Difference (LSD) were used as appropriate, with a value *P* < 0.05 considered significant for mean differences using STATGRAPHICS® Centurion XVII software.

## Results

### *n*-Butanol alters salicylic acid related genes transcription

**Figure 1:**
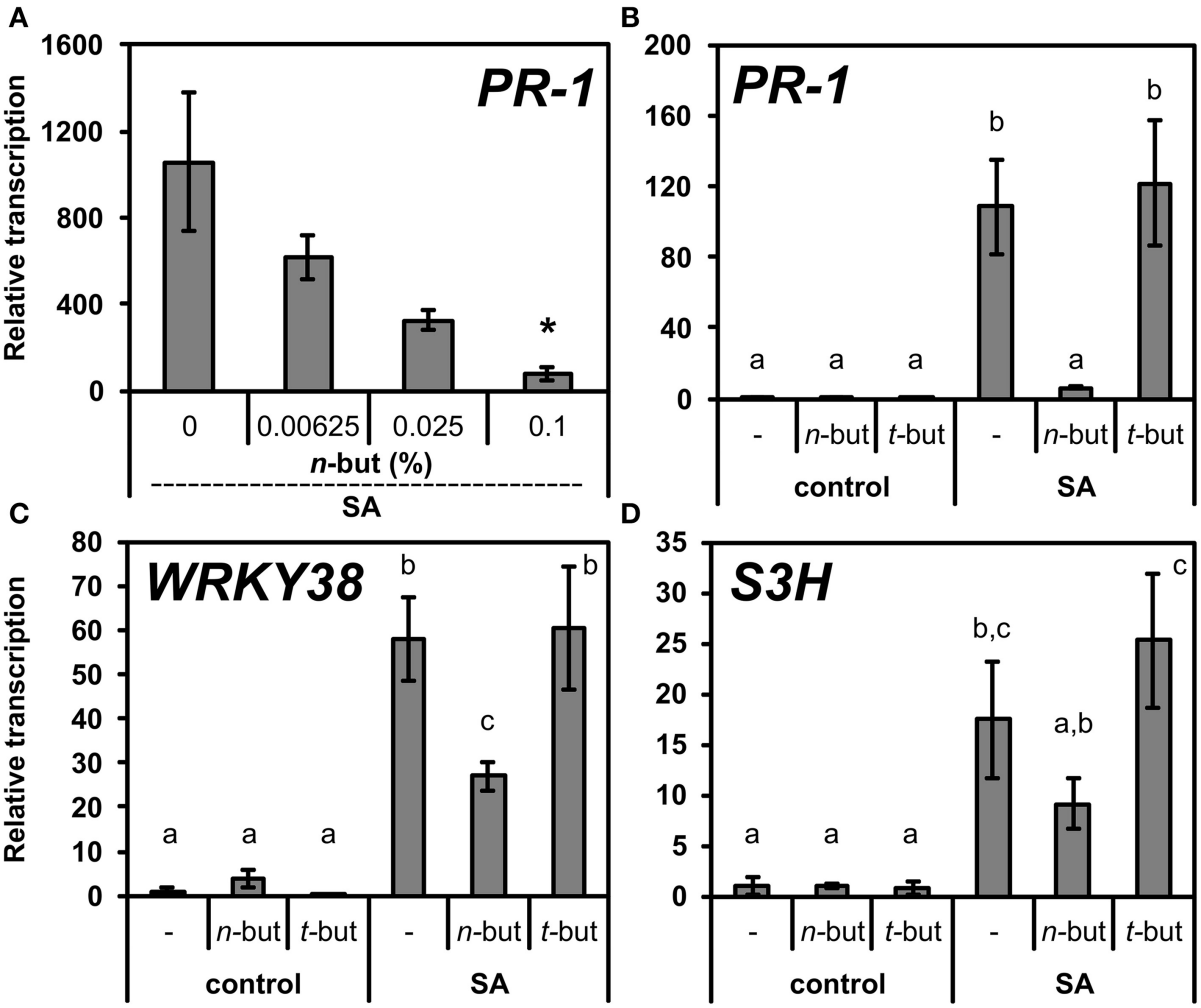
**Effect of *n*-butanol on SA related genes transcription. (A)** Ten-day-old *A. thaliana* seedlings were treated for 6 h with 50 μM NaSA (SA) and 0.00625, 0.025, 0.1% *n*-butanol. **(B–D)** 10-day-old *A. thaliana* seedlings were treated for 6 h with 0.1% *n*-butanol and 0.1% *t*-butanol or with 50 μM NaSA together with the above mentioned alcohols. Pure MS was used as a control. Error bars represent SE from three biological repeats. Asterisks indicate statistically significant differences compared to NaSA-treated plants without *n*-butanol (^*^P < 0.05, Student's *t*-test) for **(A)**. Different letters indicate significant differences (*P* < 0.05) and were calculated with One-Way ANOVA and Fisher's LSD test. Transcription was normalized to a reference gene *SAND*.

The increased levels of SA or exogenous treatment with this phytohormone activates the signaling pathway resulting in the transcription of defense related genes (e.g., pathogenesis related). The generally accepted marker of SA signaling is the *PR-1* gene. In order to examine possible role of PLD/PA in this process, we co-treated 10-day-old seedlings of *A. thaliana* with both SA and increasing concentrations of *n*-butanol. The SA induced *PR-1* transcription was decreased in the presence of *n*-butanol in a strongly dose-dependent manner (Figure 1A). Contrarily, the *t*-butanol showed no effect on the *PR-1* transcription (Figure 1B).

We also examined the effect of both alcohols on the transcription of other SA related genes, *WRKY38* and *S3H*, both encoding proteins with different functions. While the PR-1 protein is responsible for a direct antimicrobial effect as the end product of SA pathway, WRKY38 is a transcription factor which negatively regulates *PR-1* transcription, but it is NPR1 dependent (Kim et al., 2008). S3H is an enzyme responsible for the conversion of SA to a less biologically active compound, gentisic acid (Zhang et al., 2013). *WRKY38* and *S3H* transcriptions were not as significantly blocked as *PR-1* transcription. The relative transcription of *WRKY38* decreased only two times and even less in the case of *S3H* (Figures 1B–D). Also, the dose dependence of the *n*-butanol effect on the transcription of these two genes was far less apparent (Supplemental Figure S1).

### *n*-Butanol affects NPR1 accumulation in nucleus

**Figure 2:**
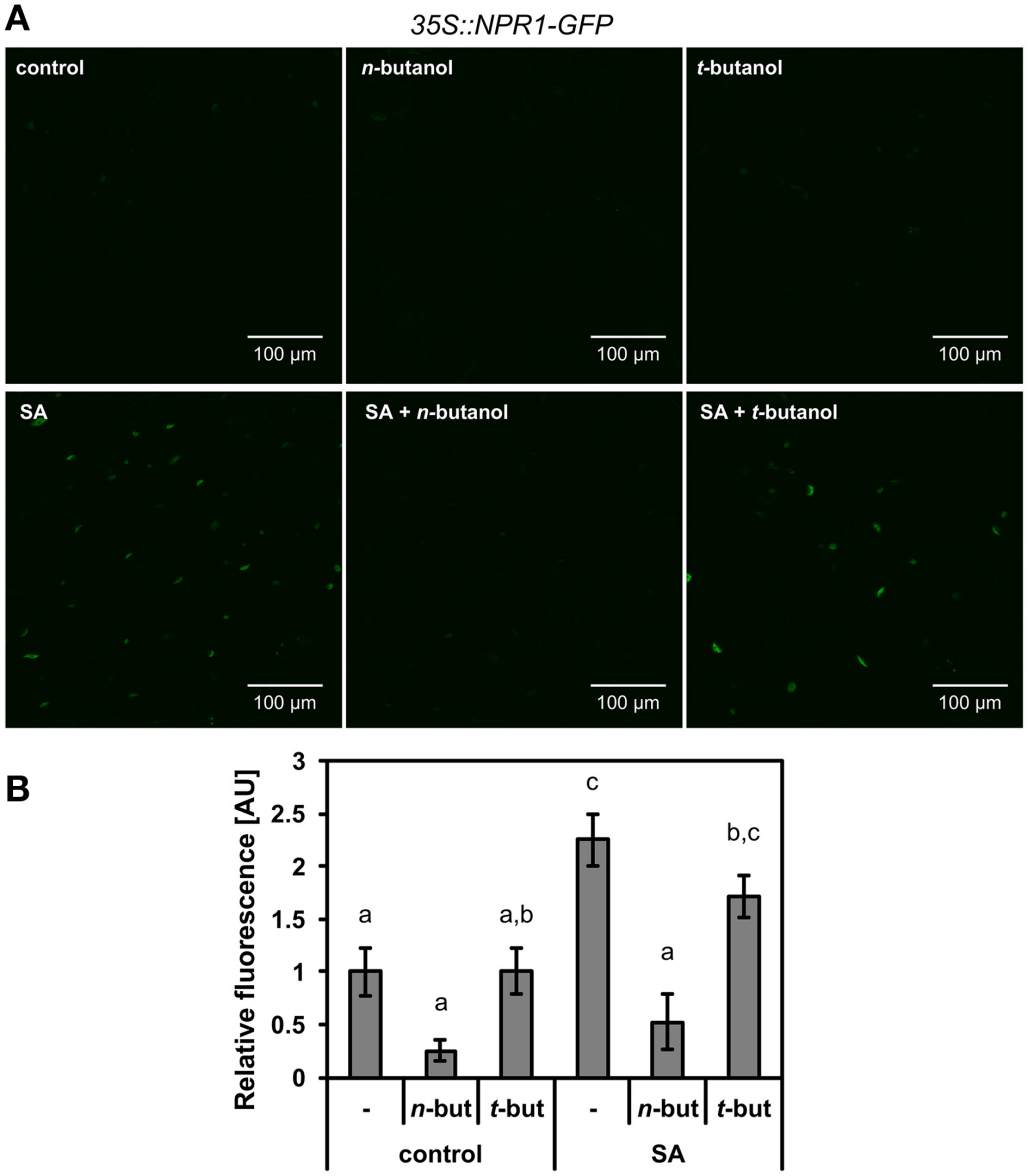
**Effect of SA and *n*-butanol on the localization of NPR1**. Ten-day-old seedlings of *A. thaliana 35S::NPR1-GFP* mutants were treated for 6 h with fresh MS medium (control), 1% *n*-butanol (*n*-but), 1% *t*-butanol (*t*-but), 250 μM NaSA (SA), 250 μM NaSA (SA) and 1% *n*-butanol or 1% *t*-butanol. **(A)** Representative micrographs of *35S::NPR1-GFP A. thaliana* seedlings 6 h after treatment, **(B)** Image analysis of relative fluorescence using APS Assess 2.0 software. The values represent means ± SE from 16 images (8 seedlings). Different letters indicate significant differences (*P* < 0.05) and were calculated with One-Way ANOVA and Fisher's LSD test. The experiment was performed in three biological replicates with similar results.

**Figure 3:**
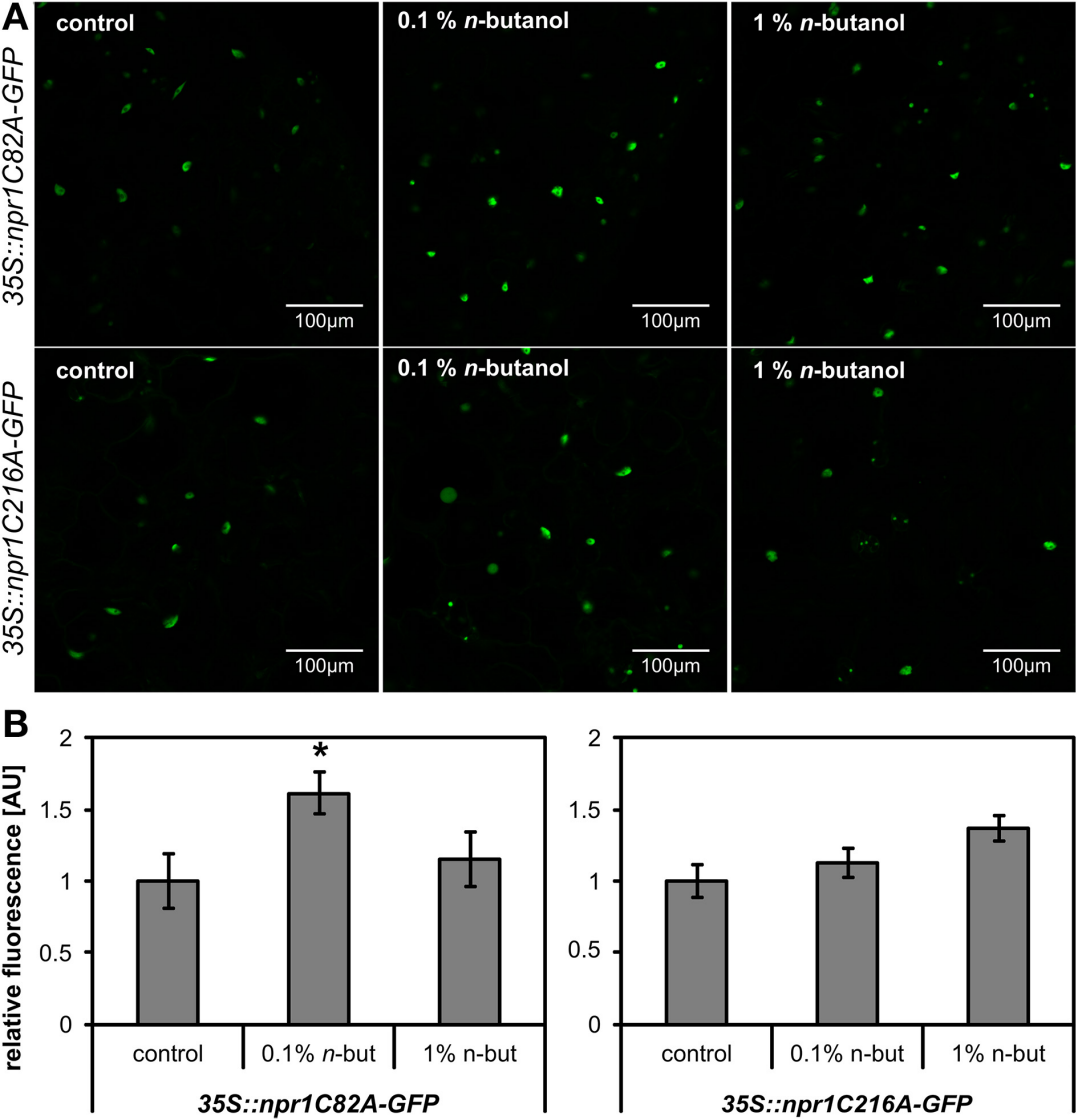
**Effects of *n*-butanol on the accumulation of NPR1 in the nuclei of *Arabidopsis thaliana* mutants constitutively expressing monomers of NPR1**. Ten-day-old seedlings of *35S::npr1C82A-GFP* and *35S::npr1C216A-GFP A. thaliana* mutants were treated 6 h with fresh MS medium (control), 0.1% *n*-butanol and 1% *n*-butanol (*n*-but). **(A)** Representative images of *35S::npr1C82A-GFP* and *35S::npr1C216A-GFP A. thaliana* seedlings 6 h after treatment, **(B)** Image analysis of relative fluorescence using APS Assess 2.0 software. The values represent means ± SE from 12 images (6 seedlings). Asterisks indicate statistically significant differences compared to the control, non-treated plants (^*^P < 0.05, Student's *t*-test). The experiment was performed in two biological replicates with similar results.

We further intended to take a closer look at the site of *n*-butanol action in the SA signaling pathway. To decipher, we used *35S::NPR1-GFP A. thaliana* transgenic plants. It was confirmed earlier that the treatment of these mutants with 2,6-dichloroisonicotinic acid (INA), a functional analog of SA, causes monomerization of NPR1, which is afterwards accumulated in the plant cell nucleus (Mou et al., 2003). We treated 10-day-old *35S::NPR1-GFP A. thaliana* seedlings with 250 μM NaSA and observed a significant increase of fluorescence in the nuclei (Figures 2A,B), the same effect was described for INA treatment. The accumulation of *35S::NPR1-GFP* in the nuclei in the presence of NaSA decreased after addition of 1% *n*-butanol (Figures 2A,B). When *t*-butanol was applied as a negative control, no effect on the *35S::NPR1-GFP* accumulation in the nuclei was observed (Figures 2A,B). *n*-butanol alone decreased the basal accumulation of NPR1 in the nuclei in the control plants, while no effect was observed for *t*-butanol. The localization of NPR1-GFP in the nuclei was verified by DAPI staining (Supplemental Figure S2). All these results correlate with the aforementioned *PR-1* transcription analysis (Figure 1B). Consequently, we wanted to examine whether the decreased amount of NPR1 in the nucleus caused by *n*-butanol is due to the higher actvity of proteasomes in NPR1 degradation (Spoel et al., 2009). For this experiment, we used *35S::npr1C82A-GFP* and *35S::npr1C216A-GFP* seedlings expressing constitutively monomerized NPR1, which is overaccumulated in the nucleus (Mou et al., 2003). The treatment of these mutants with 0.1% and 1% *n*-butanol did not decrease the accumulation of NPR1 in the nuclei (Figure 3). This experiment also provides evidence that *n*-butanol does not influence fluorescence intensity.

### *n*-Butanol induces *ICS1* transcription

**Figure 4:**
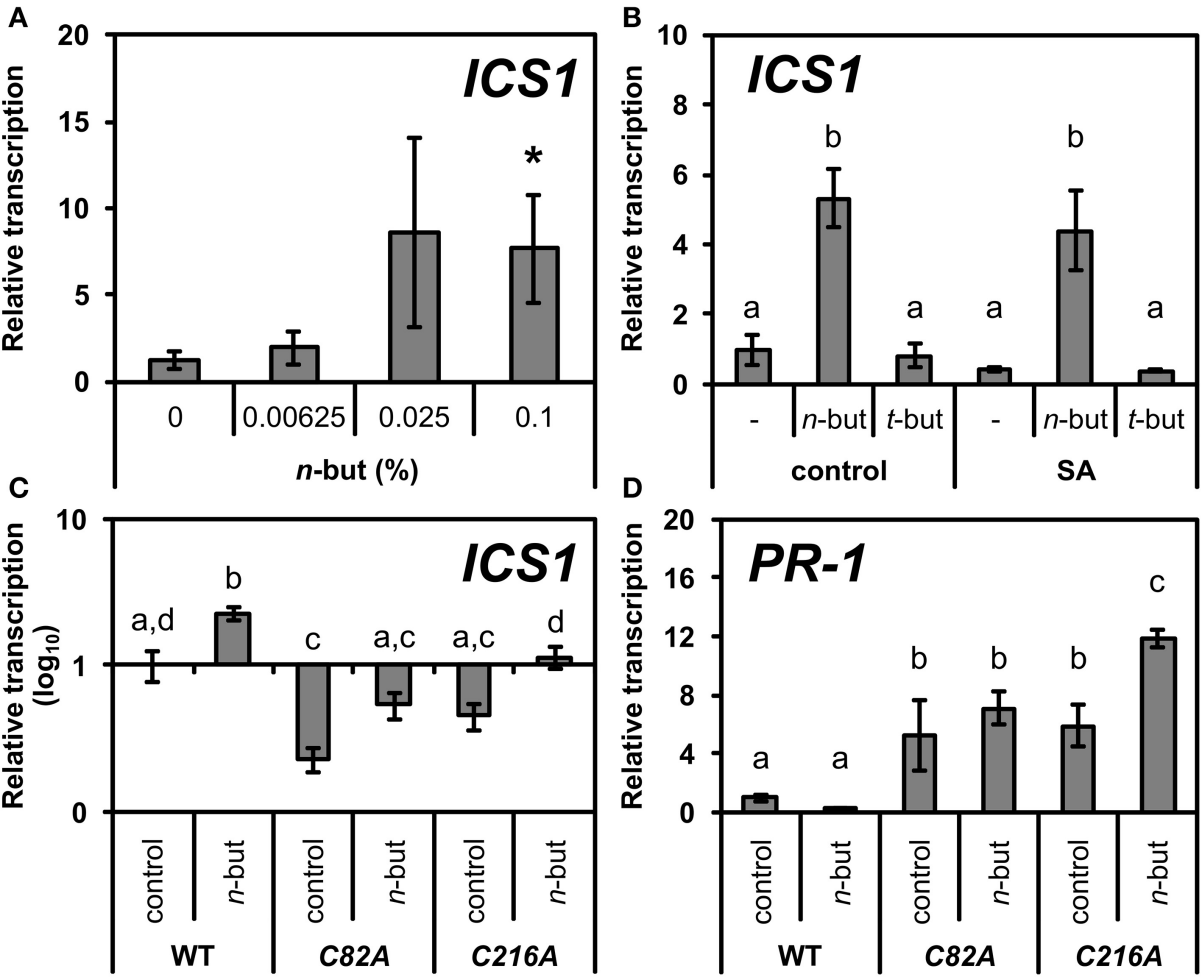
**Transcription of *ICS1* and *PR-1* in wild type and mutants of *Arabidopsis thaliana* constitutively expressing monomeric NPR1 in the presence of *n*-butanol. (A)** Ten-day-old *A. thaliana* seedlings (wt) were treated for 6 h with 50 μM NaSA (SA) and 0.00625, 0.025, 0.1% *n*-butanol. **(B)** Ten-day-old *A. thaliana* seedlings (wt) were treated for 6 h with 0.1% *n*-butanol and 0.1% *t*-butanol or with 50 μM NaSA (SA) together with the above mentioned alcohols. **(C**,**D)** Ten-day-old seedlings of *35S::npr1C82A-GFP (C82A)* and *35S::npr1C216A-GFP (C216A) A. thaliana* mutants were treated for 6 h with fresh MS medium (control) or MS with 0.1% *n*-butanol. Error bars represent SE from three independent repeats. Asterisks indicate statistically significant differences compared to control, non-treated plants (^*^P < 0.05, Student's *t*-test) for **(A)**. Different letters indicate significant differences (*P* < 0.05) and were calculated with One-Way ANOVA and Fisher's LSD test for **(B–D)**. The *ICS1* and *PR-1* transcription was normalized to a reference gene *SAND*.

Zhang et al. (2010) showed that nuclear localization of NPR1 is required for SA accumulation, *ICS1* transcription and SA tolerance. When NPR1 was retained in the cytoplasm, plants accumulated higher levels of *ICS1* transcripts compared to the wild type. Based on this, we measured the transcription of *ICS1* upon the addition of the *n*-butanol treatment and as expected, *n*-butanol induced *ICS1* transcription in a dose-dependent manner (Figure 4A), while *t*-butanol had no effect (Figure 4B). These results support our suggestion that *n*-butanol inhibits the translocation of NPR1 to the nucleus. Accordingly, we also found that the *ICS1* transcription in *35S::npr1C82A-GFP* and *35S::npr1C216-GFP* mutants were significantly decreased but *n*-butanol treatment partially reverted this effect (Figure 4C).

### *n*-Butanol causes changes in *A. thaliana* metabolome

As the accumulation of SA leads to the massive reprogramming of the plant transcriptome, it was obviously accompanied by significant changes in the whole metabolome. We investigated these changes in plants treated with SA and *n*-butanol to test if we could reveal compounds involved in the SA/phospholipid signaling pathway.

**Figure 5:**
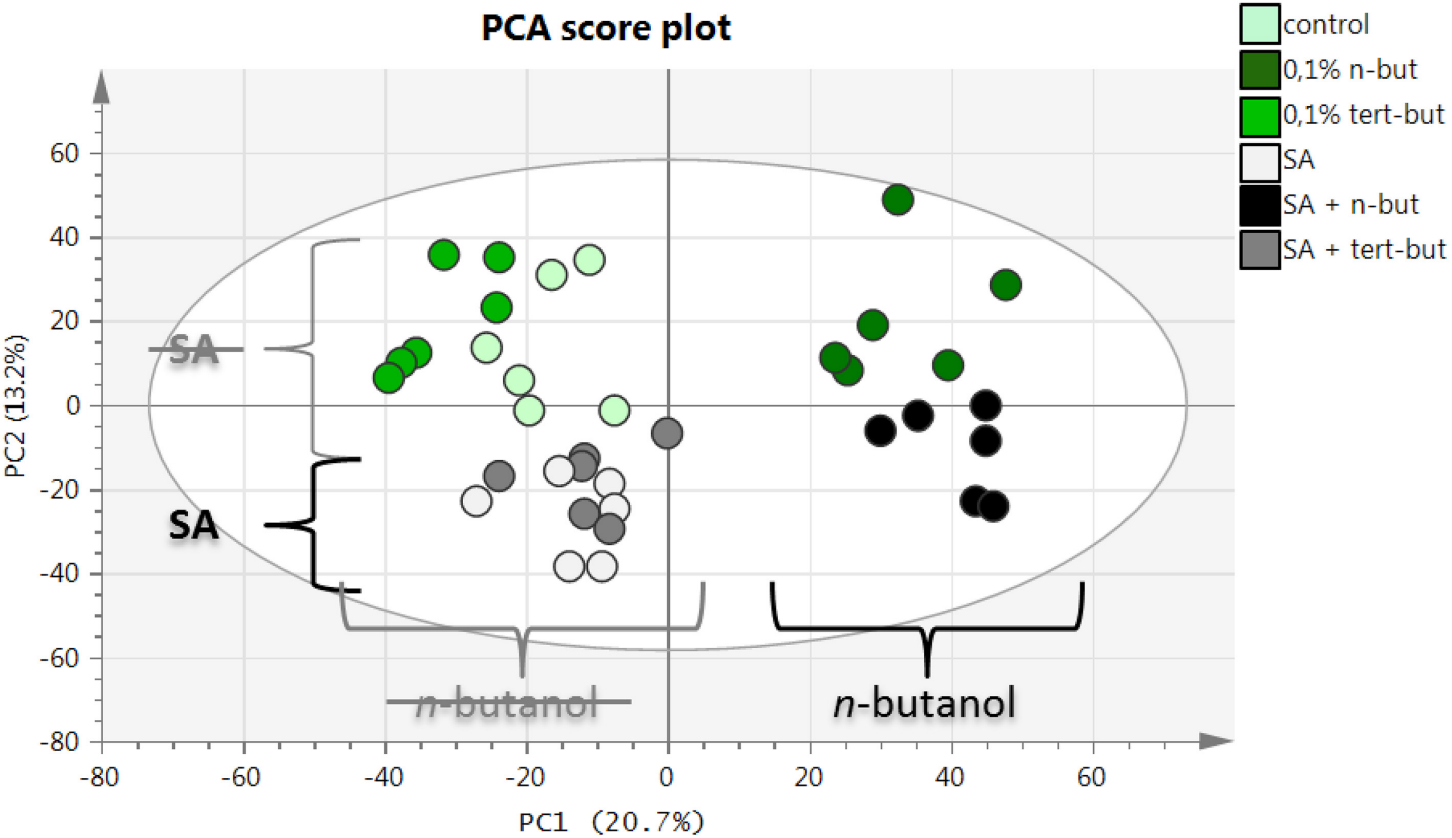
**PCA score plot for LC-ESI(-)-MS data of *n*-butanol and salicylic acid metabolome in *A. thaliana***. Ten-day-old *A. thaliana* seedlings were treated for 6 h with 0.1% *n*-butanol or 0.1% *t*-butanol or with 50 μM NaSA (SA) together with the aformentioned alcohols. Fresh MS medium was used as a control. This experiment was done in three biological repeats with similar results.

The principle component analysis (PCA) represents a highly useful and widely employed tool for the interpretation of complex data sets generated by several modern instruments including mass spectrometry. In our study, PCA was employed to explore alterations in the metabolomes of differently treated *A. thaliana* samples measured by LC-MS. As shown in Figure 5, there was a significant difference in the metabolomic fingerprints of samples treated with *n*-butanol (right side of the PCA plot) and untreated ones (left side of the PCA plot). The samples treated with SA were clearly differentiated (bottom part of the PCA plot) from the untreated samples (top part of the PCA plot). The list of the most distinct markers (ions recovered from the LC-MS records) is summarized in Table 1. Obviously, SA and its metabolite SA hexoside are typical markers for the samples treated with SA. Unfortunately, the identification of markers present in *n*-butanol treated plants was mostly unsuccessful, mainly due to their high *m/z* values resulting in many possible elemental formulas and also due to the limited information about the changes induced by *n*-butanol in the metabolism. Important observations were that the *t*-butanol treated samples did not differentiate from the untreated samples and also that *t*-butanol had no effect on the SA treated samples (Figure 5).

**Table 1:** **The most distinct metabolites (markers) of *A. thaliana* seedlings**.

**m/z**	**RT (min)**	**Ion elemental Formula**	**Tentative identification**	**Ion**	**Mass error (ppm)**	**Marker of treatment**
137.0240	3.31	C_7_H_5_O_3_	Salicylic acid	[M-H]-	5.0	SA
202.0714	1.61	C_8_H_12_NO_5_	N-Butyryl-L-aspartic acid	[M-H]-	2.0	n-but
235.1180	2.63	C_10_H_19_O_6_	Butyl—hexoside	[M-H]-	1.6	n-but
295.1028	1.79	C_11_H_19_O_9_	?	?	1.5	n-but
299.0768	2.04	C_13_H_15_O_8_	Salicylic acid—hexoside	[M-H]-	2.2	SA
536.1651	0.69	?	?	?	?	n-but
643.2446	2.63	?	?	?	?	n-but

**Figure 6:**
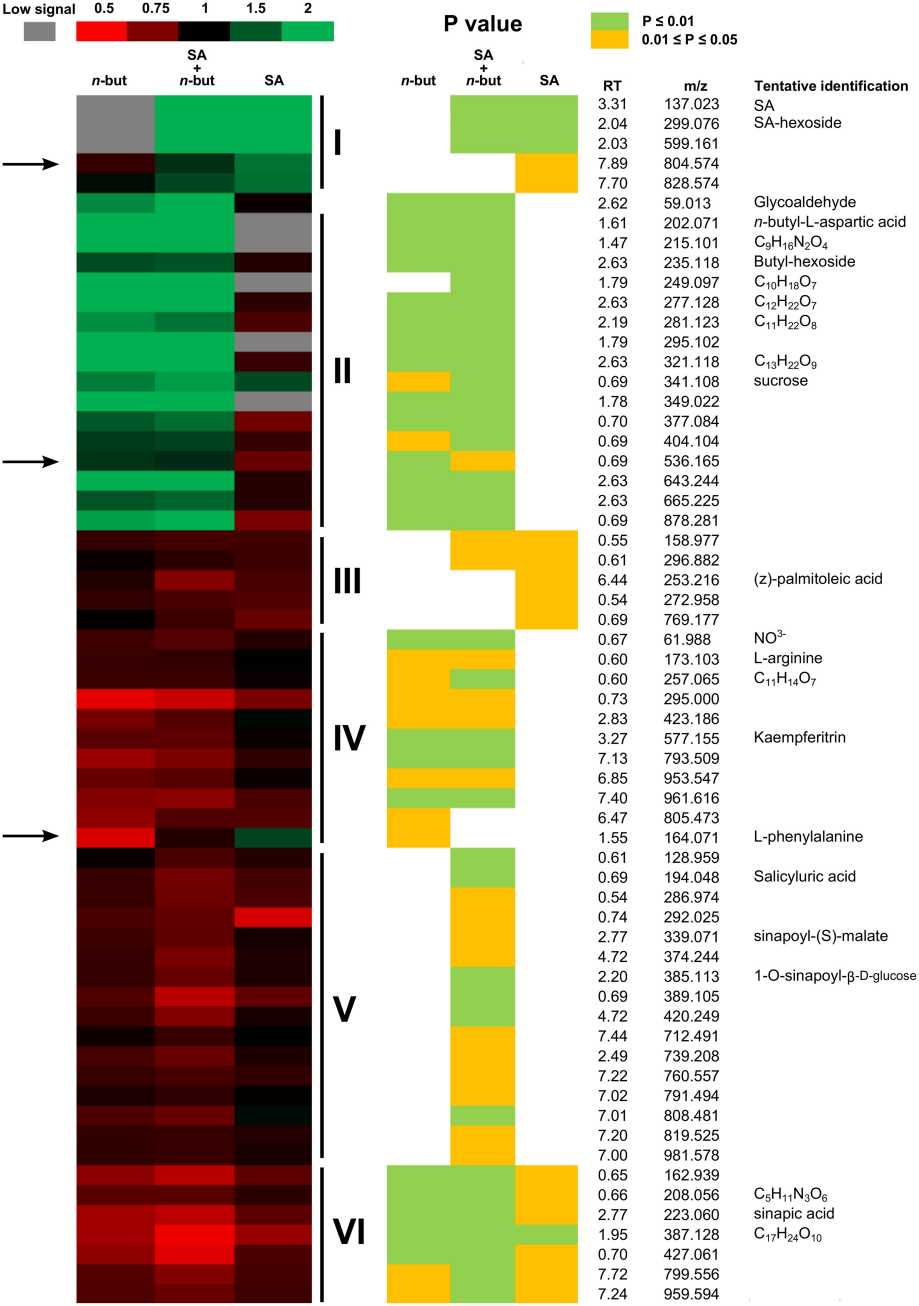
**Heat map of metabolites**. Ten-day-old *Arabidopsis thaliana* seedlings were treated for 6 h with 0.1% *n*-butanol or with 50 μM NaSA (SA) and both chemicals together. Fresh MS was used as a control. The Heat map values represent a ratio between the treated and control samples (treatment/control). The green color indicates increased values, red indicates decreased values and black indicates zero; see the color scale. The gray color indicates metabolites with a low signal in particular treated samples. *P*-value is represented by yellow 0.01 < *P* < 0.05; bright green *P* < 0.01 (Student's *t*-test). The arrows indicate putative metabolites with a similar pattern of response to treatment as the *PR-1* gene transcription. Group I represents metabolites induced by SA. Group II represents metabolites induced by *n*-butanol. Group III represents metabolites suppressed by SA. Group IV represents metabolites suppressed by *n*-butanol. Group V represents metabolites suppressed only when SA and *n*-butanol were applied together. Group VI represents metabolites suppressed by all treatments. This experiment was repeated in three biological repeats with similar results. RT, retention time; m/z, mass to charge ratio.

We identified 114 metabolites, from which 61 were statistically (*P* = 0.05; two tailed Student's *t*-test) changed at least in one of the used treatments (Figure 6). Based on the response to treatment, we were able to divide the metabolites into six groups (Figure 6) according to whether they were induced or suppressed by SA, *n*-butanol or both chemicals together.

## Disscussion

Plant response to biotic stress mediated by the phytohormone SA is a fundamental process. It was shown that NPR1 protein is a crucial component of the SA signaling (Cao et al., 1994). The structural changes and localization of this protein in plant cells is responsible for the plant defense signaling (Kinkema et al., 2000). Whereas an oligomer form occurs in cytosol, the monomer, which is formed when SA level increases, is translocated to the nucleus (Mou et al., 2003), where it binds to the TGA transcription factors and induces a transcription of the most of SA related genes (Zhang et al., 1999; Wang et al., 2005). NPR1 nuclear localization is responsible for regulation of plant tolerance to SA, a negative regulation of *ICS1* transcription and leads to SA accumulation (Zhang et al., 2010). Thus, NPR1 is really a master regulator of the SA signaling pathway although the NPR1 independent pathway also exists (Janda and Ruelland, 2014). Nevertheless, there are still gaps in the knowledge of the regulation of SA signaling needing to be filled in.

### *n*-Butanol and NPR1 dependent SA signaling pathway

In our study, we have shown that *n*-butanol is a molecule with a high impact on the SA signaling pathway in *A. thaliana* seedlings. *n*-butanol has been for a long time accepted by the “PLD community” as a modulator of PLD activity due to its preference for primary alcohols as substrates (Yang et al., 1967; Munnik et al., 1995). Potocky et al. (2014) recently provided excellent evidence that *n*-butanol alters the concentration of PA on the pollen tube's plasma membrane *in vivo*. *n*-butanol was used to establish the PLD/PA signaling connection with G proteins, ABA triggered germination, primary root elongation, hypocotyl length, cotyledon expansion, inhibition of pollen tube germination and growth, proline accumulation, actin cytoskeleton rearangement and microtubule reorganization (Munnik et al., 1995; Ritchie and Gilroy, 1998; Dhonukshe et al., 2003; Gardiner et al., 2003; Potocky et al., 2003; Thiery et al., 2004; Motes et al., 2005; Pleskot et al., 2010, 2014). We would like to mention that it is necessary to keep in mind the possibility that the effect of *n*-butanol is not so specific as was mentioned by Hirase et al. (2006), who observed that *n*-butanol induced the depolymerization of microtubules. Although the use of *t*-butanol, as a control, can serve as convincing proof.

In our study, the treatment of *A. thaliana* seedlings with *n*-butanol rapidly decreased *PR-1* transcription in the presence of SA and this effect is clearly dose dependent (Figures 1A,B). The effect on the transcription of *WRKY38* and *S3H* was much less pronounced (Figures 1C,D) but in the case of *WRKY38* the decrease was significant (more than two times) and so the transcription pattern seems similar to *PR-1*. It is not surprising, as the transcription of *WRKY38* is also NPR1 dependent. Our results are in agreement with the results obtained by Krinke et al. (2009) in *A. thaliana* suspension cells. *n*-butanol did not have a significant effect on *S3H* transcription. S3H is responsible for a conversion of SA, therefore its transcription should be induced immediately by higher levels of SA and the signaling events downstream to SA can have only a minor effect on *S3H* transcription (Figure 1D). In fact, the connection between the S3H effect and NPR1 has not yet been described in detail.

A deeper insight into the mode of action of *n*-butanol in SA signaling provided the experiment with *35S::NPR1-GFP A. thaliana* mutants. We observed that effect of *n*-butanol is closely connected with the NPR1 localization in plant cells. In the *35S::NPR1-GFP* plants, *n*-butanol blocks NPR1 accumulation in the nucleus in the presence of SA (Figure 2). This finding well corresponds with the suppressive effect of *n*-butanol on the transcription of *PR-1*. Zhang et al. (2010) showed that nuclear localization of NPR1 negatively regulates the transcription of the *ICS1* gene. We observed that *n*-butanol induces the transcription of *ICS1* (Figure 4), which supports the idea that *n*-butanol blocks translocation of monomeric NPR1 to the nucleus. It was reported that the proteasome degrades NPR1 monomers in the nucleus (Spoel et al., 2009). Based on that fact we used *35S::npr1C82A-GFP* and *35S::npr1C216A-GFP* mutants which constitutively express a higher amount of NPR1 monomers and also exhibit a higher accumulation of NPR1 in the nucleus (Mou et al., 2003). In these mutants, we investigated the effect of *n*-butanol. As *n*-butanol treatment revealed no effect on the nuclear localization of NPR1 in the *35S::npr1C82A-GFP* and *35S::npr1C216A-GFP* mutants (Figure 3), we can assume that *n*-butanol acts in the cytosol in the SA pathway before or during NPR1 translocation to the nucleus. *n*-butanol could either affect the transmission of the monomer from the cytosol to the nucleus e.g., by direct effect of *n*-butanol on the nucleopores or by active transport which can be mediated by PA. This mechanism was recently shown in the nuclear localization of the MYB transcription factor (Yao et al., 2013). Nevertheless, we cannot exclude the possibility that *n*-butanol acts upstream to NPR1 monomerization (**Figure 8**).

### Phospholipids in SA signaling

Based on the above mentioned observations, we would like to highlight the possible connection between the phospholipid signaling system and the SA pathway. SA treatment increased the PA level or the PLD activity in *A. thaliana, B. napus*. and soybean (Profotova et al., 2006; Kalachova et al., 2012, 2013; Rainteau et al., 2012). Krinke et al. (2009) showed in *A. thaliana* suspension cells that SA treatment led to a rapid increase of the PA level *in vivo*. We observed that the exogenous PA is capable of preventing the disruption of the actin cytoskeleton caused by SA (Matouskova et al., 2014). PLD and PA are not the only members of large phospholipid family involved in SA signaling. Interestingly, it was shown that SA treatment activates type-III phosphatidylinositol-4-kinase (PI4K), which is responsible for the formation of phosphatidylinositol-4-phosphate (PI4P) and phosphatidylinositol-4,5-bisphosphate (PIP_2_) in *A. thaliana* suspension cells (Krinke et al., 2007). Recently, we showed that double knock-out mutation of two isoforms PI4Kβ1β2 triggers SA signaling, suggesting them to be negative regulators of SA signaling (Sasek et al., 2014). For more information about the connection between hormones and phospholipid signaling see the review (Janda et al., 2013).

**Figure 7:**
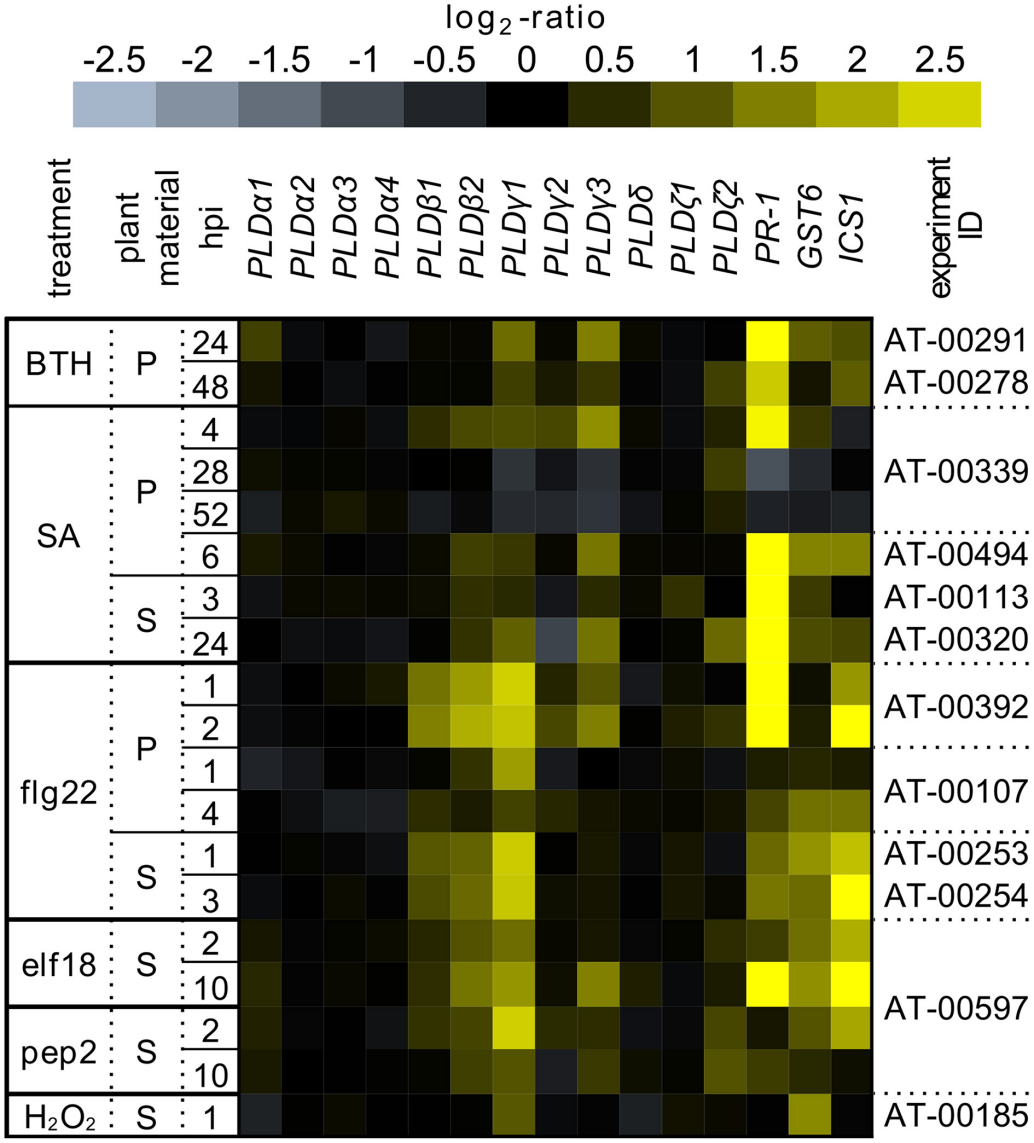
**The transcription pattern of PLDs in *A. thaliana* under stress conditions**. The transcriptomic data collected from the public database Genevestigator after treatment by BTH, SA, flg22, elf18, pep2, and H_2_O_2_. All experiments were performed on the *A. thaliana* ecotype Col-0 in the different developmental stages treated with different concentrations of compounds and in different time points. The experiment ID provides the identification for the particular experiment in Genevestigator. For comparison, the transcription of *PR-1, GST6*, and *ICS1* genes were added.

The interest of importance is to find out the particular isoform(s) of PLD responsible for the effect of *n*-butanol. Based on this, we performed the *in silico* experiment to find the possible PLD isoforms involved in SA signaling (Figure 7). We investigated the transcription of all PLD isoforms in the publicly available database, Genevestigator (Hruz et al., 2008), in response to SA, BTH (benzothiadiazole; a functional analog of SA), EF-Tu, flg22, pep2 (well described PAMPs) triggering SA signaling. All studies, which we included to this analysis, were performed on the *A. thaliana* ecotype Col-0. The screening showed that the promising candidates could be PIP_2_-dependent isoforms from the PLDβ, PLDγ and PLDζ families (Figure 7). We can speculate that they could be connected with the above mentioned PI4K activity, producing precursor for PIP_2_ biosynthesis. The evidence that PLD isoforms exhibit redundant effect upon *Pseudomonas syrigae* infection was provided recently by Johansson et al. (2014).

### Metabolomic screening

The aim of this part of our study was to find the metabolic compounds, which are affected by *n*-butanol and involved in the SA pathway. For this purpose, we used the mass spectrometry-based metabolomic fingerprinting described by Vaclavik et al. (2013). A very important output from the screening is the evidence that *t*-butanol treatment, used in our study (but also by other researchers studying PLD function) as a negative control to *n*-butanol, is really biologically “inactive.” This is based on the fact that the PCA analysis of the samples determined no differences between control samples vs. *t*-butanol and the SA treated samples vs. *t*-butanol (i.e., their metabolomes were similar). On the other hand, the samples treated with *n*-butanol clustered very well (Figure 5). We were able to identify several characteristic metabolites for the samples treated with *n*-butanol. We were also able to predict the molecular formulas and we proposed their tentative identification (Table 1). It is not surprising that a higher amount of SA and SA-hexoside was found in the samples treated by SA. In fact, we were able to identify only a few metabolites affected by SA, probably due to the relatively short time of treatment. It was shown that BTH causes significant alterations in metabolome 24 h after treatment, while after 4 h the changes were much less significant (Hien Dao et al., 2009). We chose the 6 h time-point to get the information in the same time frame as we used for the transcriptional study (Figure 1). Interestingly, *n*-butanol had a higher impact on the *A. thaliana* metabolome compared to the SA treatment. Seventeen metabolites were affected by SA and 34 by *n*-butanol (Figure 6).

We were able to predict structure for several metabolites that changed upon treatment. The heat map representing the changes of 61 metabolites supplemented with RT-m/z and the putative names of several predicted compounds is shown in Figure 6. Interestingly, the behavior of phenylalanine, a precursor of SA biosynthesis, exhibits a similar pattern as *PR-1* transcription upon treatment. Another two compounds exhibit patterns similar to *PR-1* (RT_m/z 7.89_804.5 and 0.69_536.1; Figure 6). These compounds could be interesting targets of further research.

## Conclusion

**Figure 8:**
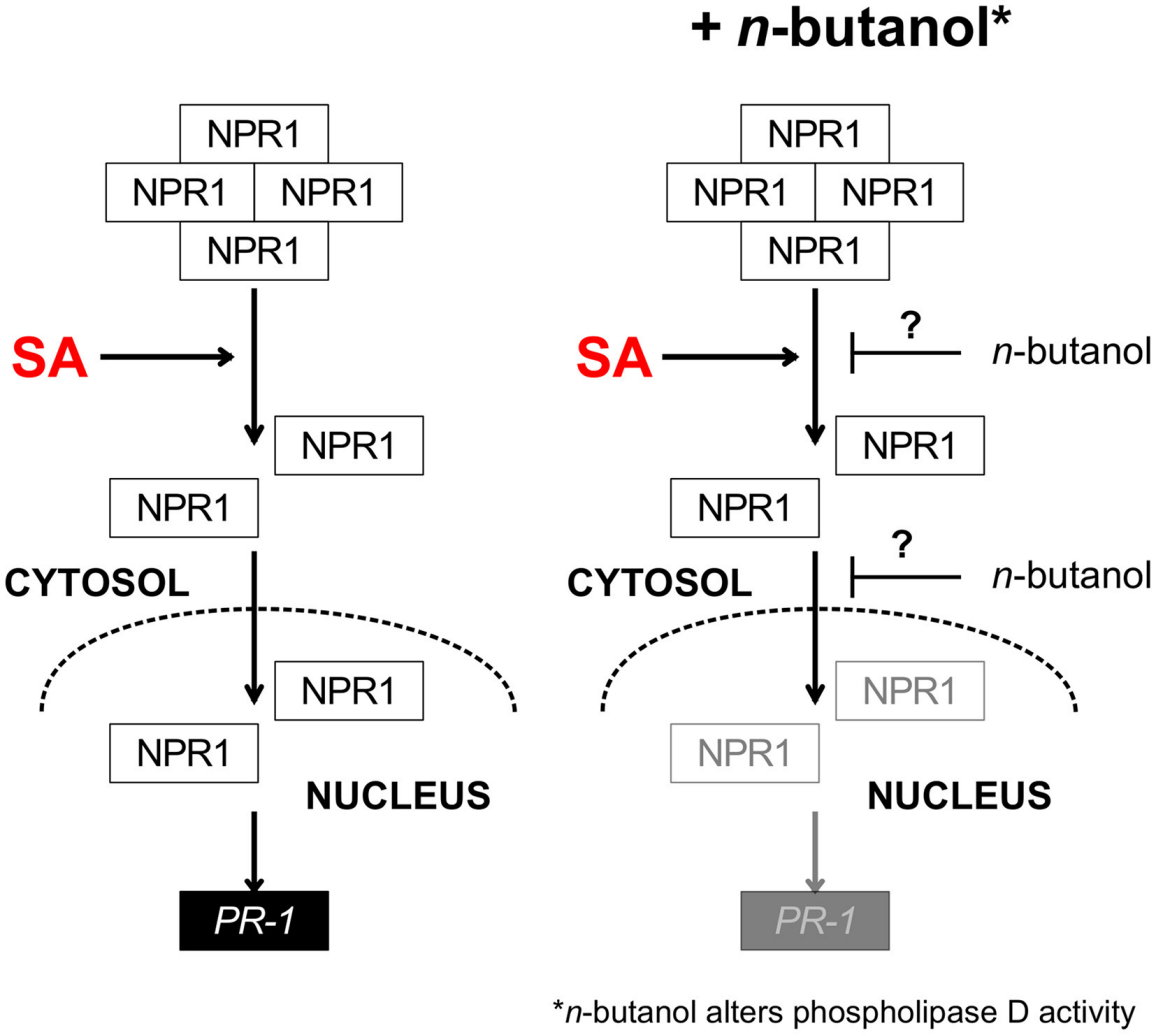
**Scheme summarizing the effect of *n*-butanol on the SA pathway in *A. thaliana***. In the presence of salicylic acid NPR1 monomerize, translocates into the nucleus and induces the *PR-1* gene transcription. In the presence of *n*-butanol the accumulation of NPR1 in the nucleus is decreased and the transcription of *PR-1* is supressed. The proposed site of *n*-butanol effect is shown by blunt-end arrows with question mark. SA—salicylic acid, NPR1—nonexpressor of pathogenesis related1, *PR-1*—pathogenesis related 1.

The observations were summarized in the scheme presented in Figure 8. *n*-butanol affects *PR-1* transcription and NPR1 accumulation in the nucleus in the presence of SA. We propose that our current study should be a new puzzle fitting in the previous idea that PA produced by PLD is involved in the SA signaling pathway as *n*-butanol alters PLD activity. We found 61 metabolites whose levels were changed upon the treatment with *n*-butanol and SA. We showed that the *n*-butanol treatment has a higher impact on the metabolome than treatment with SA. We provided the metabolomic evidence that *t*-butanol can be really used as a negative control in studies using *n*-butanol.

## Author contributions

Martin Janda created the conception and design, performed and analyzed the experiments and also composed the manuscript. Vladimír Šašek designed, performed and analyzed the experiments. Jan Andrejch performed and analyzed the confocal microscopy experiments. Hana Chmelařová performed and analyzed the experiments (metabolomic screening). Miroslava Nováková performed and analyzed the experiments (confocal microscopy) and critically revised the manuscript. Jana Hajšlová analyzed the data (metabolomic screening) and critically revised the manuscript. Lenka Burketová critically revised the manuscript. Olga Valentová created the conception, critically revised the manuscript and also composed the manuscript. All authors concurred in the final version of the manuscript.

### Conflict of interest statement

The authors declare that the research was conducted in the absence of any commercial or financial relationships that could be construed as a potential conflict of interest.

## Abbreviations

NPR1: nonexpressor of pathogenesis related 1
PA: phosphatidic acid
PDK1: 3-phosphoinositide-dependent protein kinase 1
PI4K: phosphatidylinositol 4-kinase
PI4P: phosphatidyl inositol 4-phosphate
PIP_2_: phosphatidylinositol 4,5-bisphosphate
PR: patogenesis related
PLD: phospholipase D
SA: salicylic acid.
