# Supplementary material for: Phospholipase D affects translocation of NPR1 to the nucleus in Arabidopsis thaliana
Source: Front Plant Sci. 2015 Feb 18;6:59. doi: 10.3389/fpls.2015.00059 (PMC4332306; doi:10.3389/fpls.2015.00059)
Supplement: Supplementary file 1 [file Image1.PDF]

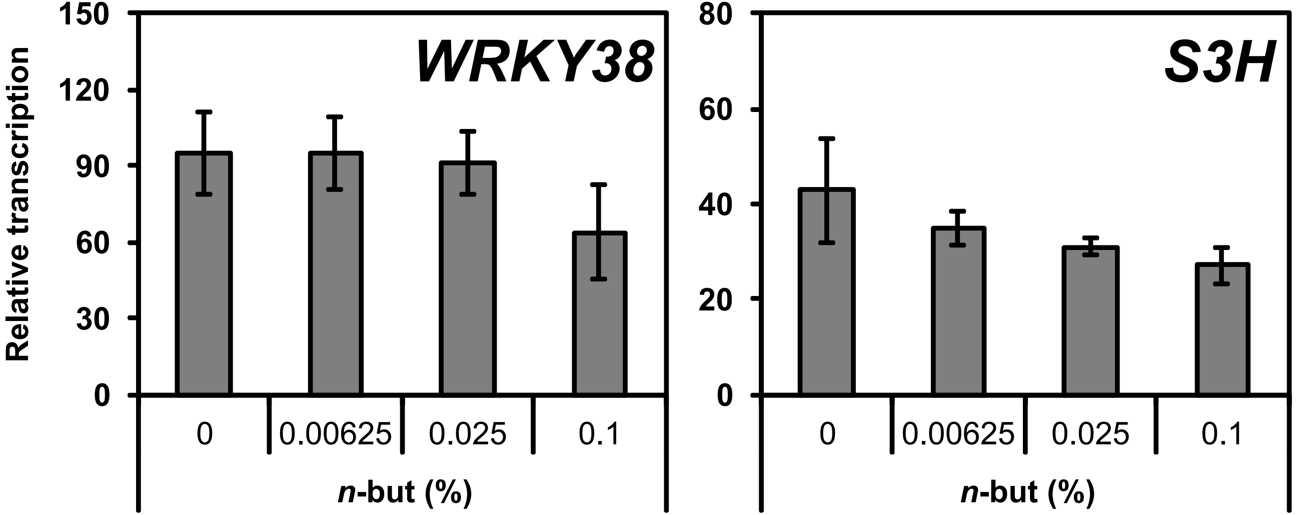


**Figure S1. Dose dependent effect of *n*-butanol on the transcription of *WRKY38* and *S3H*.** 10 days old *A. thaliana* seedlings were treated for 6 h with 50 µM NaSA (SA) and 0.00625, 0.025, 0.1 % *n*-butanol. The *WRKY38* and *S3H* expressions were normalized to a reference gene *SAND*.
