# Supplementary material for: Phospholipase D affects translocation of NPR1 to the nucleus in Arabidopsis thaliana
Source: Front Plant Sci. 2015 Feb 18;6:59. doi: 10.3389/fpls.2015.00059 (PMC4332306; doi:10.3389/fpls.2015.00059)
Supplement: Supplementary file 2 [file Image2.PDF]

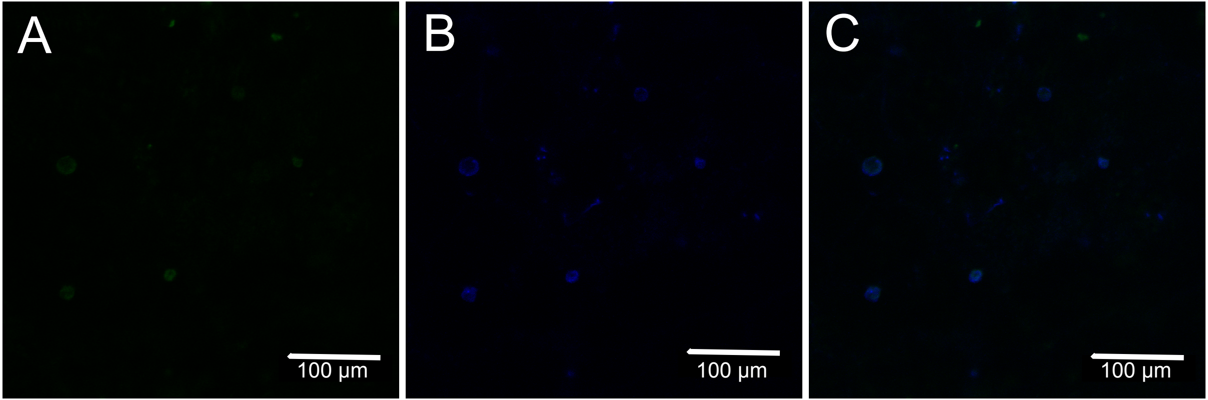

Figure S2. Nuclear localization of NPR-GFP and DAPI staining of nuclei. Representative micrographs of *35S::NPR1-GFP A. thaliana* 10-days old seedling treated for 6h with 250 µM SA. (A) Fluorescence of NPR1-GFP was excited by 488 nm line of a laser, (B) DAPI fluorescence of nuclei was excited by the 405 nm line, (C) overlap of both
